# Sleep-push movement performance in elite field hockey champions with and without training specialization

**DOI:** 10.3389/fpsyg.2023.1199448

**Published:** 2023-07-31

**Authors:** Killian Cosendey, Scott Mongold, Mathieu Petieau, Guy Cheron, Ana-Maria Cebolla

**Affiliations:** ^1^Laboratory of Neurophysiology and Movement Biomechanics, Université Libre de Bruxelles, Brussels, Belgium; ^2^Laboratory of Neuroscience, Université de Mons, Mons, Belgium

**Keywords:** field hockey player, EMG – electromyogram, kinematics and dynamics, elite performance, specialized training

## Abstract

**Objective:**

To investigate kinematic and muscle activity differences during the sleep-push movement in elite field hockey players. We hypothesized that players with specialized sleep-push movement training (specialists) would possess a lower center of mass (CoM) and enhanced reproducibility of muscle activations during the movement, compared to players without explicit movement training (non-specialists).

**Methods:**

Ten field hockey players of the Belgian national field hockey team performed the sleep-push movement (5 specialists and 5 non-specialists). Muscle activity and kinematic data were recorded using EMG to evaluate the reproducibility of muscle activations by cross-correlation analysis and power spectral features across the movement, while a motion capture system was used to assess kinematics.

**Results:**

Compared to non-specialists, specialists had significantly (*p* < 0.05) increased stick velocity (9.17 ± 1.28 m/s versus 6.98 ± 0.97 m/s) and lower CoM height (141 ± 52 mm versus 296 ± 64 mm), during the second part of the shot. Specialists also showed a significant (*p* < 0.05) lower power spectrum in the activity of the upper limb muscles before the shot. Superimposition of the auto crosscorrelation results demonstrated a high degree of reproducibility in specialists’ muscle activations.

**Conclusion:**

Sleep-push movements realized by elite players who are specialists in the sleep-push movement presented significant kinematics and muscular activation differences when compared to the sleep-push movements realized by elite players who were not specialists in such movement. Characterization of the specific movement and the related high-level performer’s muscular strategies offers the possibility of translating sport science findings into functional training with concrete applications for coaches, players, and other key stakeholders for the continued development of the field.

## Introduction

Elite performance in sports largely depends on a host of physiological factors, of which, proper motor coordination of numerous muscles is crucially important (Feldman et al., 1995; d’Avella and Lacquaniti, 2013; Ivanenko and Gurfinkel, 2018). Constant recalibration of motor strategies must be performed by the central nervous system (CNS) to achieve task success (Gaveau et al., 2014; Shadmehr, 2017). Professional athletes provide a unique opportunity to study highly practiced movements, resulting in highly coordinated motor control strategies and heightened performance compared to a non-athlete (Vanderstukken et al., 2020). In the context of sport competition, the ability to quantify performance-based differences, as they relate to neuromuscular activity and postural control, across elite professional athletes with and without specialized training, should provide insight into nuanced motor control strategy. The comparison of elite athletes of the same sport, where specialization is position-specific, may result in findings that can be integrated into coaching strategy (McGuinness et al., 2020; Goods et al., 2022).

Previous studies have analyzed field hockey through both a physiological and psychological lens, exploring biochemistry, anthropometric characteristics, or game strategy (Elferink-Gemser et al., 2007; Macutkiewicz and Sunderland, 2011, 2018; Huijgen et al., 2013; Sunderland and Edwards, 2017; Leyhr et al., 2018); For example, it has been shown that the same protocol of hamstring muscle stretching produced different ranges of movement, passive resistance to stretches, and muscular activity in elite field hockey players compared to normal subjects (Jaeger et al., 2003). Also, elite field hockey players have shown altered intramuscular and intermuscular balance ratios during maximal shoulder-girdle contractions, suggesting a sport-specific adaptation to optimize the coordinated activity of the scapulothoracic muscles (Vanderstukken et al., 2020). However, few have studied the sleep-push shot (also called drag-flick; Yussof et al., 2008; de Subijana et al., 2011; Gómez et al., 2012; Ibrahim et al., 2017; Ng et al., 2018). Therefore, kinematic information is limited (Yussof et al., 2008; de Subijana et al., 2011). This movement consists of an approach phase, shot phase, and follow-through, which occur sequentially as the player reaches the ball and swings the stick. As there are only right-handed sticks, the sleep push is performed with a similar whole-body orientation across players. The left leg is the closest to the goal and provides stability during the shot. The right leg is considered the drive leg: the leg which propels the body forward thus aiding in ball acceleration. Several studies have focused on technique improvement (de Subijana et al., 2011; Meulman et al., 2012), but none have characterized potential differences in kinematic and muscular activity, which may improve training or coaching strategies.

In sporting situations, the ability to assess neuromuscular activity is often done through EMG measurement (Barnamehei et al., 2018). Importantly, highly dynamic movements, like the sleep-push shot in field hockey, require refined control of muscles in the upper and lower extremities, in addition to postural muscles. Concerning the sleep push, task success corresponds to scoring a goal. Accuracy across shots and throughout a season is highly valued. Specialized training of the sleep push should thus foster reproducible accuracy. The capacity to reproduce the same pattern of muscle activity should be higher in those who have trained more extensively: the sleep-push specialists. Conveniently, EMG signals are good physiological indicators of the temporal structure of motor strategies. It is possible to compare the temporal component of EMG signals with cross-correlation analysis (Cheron, 2015). Cross-correlation is a method frequently used to compare muscle activations between the same or different muscles (Amblard et al., 1994; Cheron et al., 1997, 2001; Wren et al., 2006; Dorel et al., 2008; Bengoetxea et al., 2010). Furthermore, the spectral features of EMG represent additional means to quantify potential differences in muscle activity/inactivity and frequency-specific power (Filli et al., 2019).

Another critical feature in sports gestures is the ability to maintain stability. Corrective movements in response to poor balance result in additional time before the next movement can commence, and in the context of field hockey, could be the difference between an open shot or a well-defended one. CoM measurement is a common method to quantify stability (Orendurff et al., 2004), where when CoM is kept lower, excursions horizontally are minimized, leading to greater balance (Stapley et al., 1999; Mapelli et al., 2014). CoM measures are usually interpreted as indicators of postural stability in quiet standing. However, it seems inadequate to characterize the higher-level performance of athletes who are expected to show superior postural control in dynamic movements (Garcia et al., 2011; Hrysomallis, 2011; Opala-Berdzik et al., 2021). Considering this, we propose that maximizing stability during the sleep-push movement might contribute to better performance and greater shot accuracy. We aimed to assess CoM height in the vertical direction to examine stability across players.

The purpose of this study was to investigate how top field hockey athletes specializing in the sleep-push movement compare to their professional counterparts. We quantified whole-body kinematics and muscle activity to explore the difference in their performance. We hypothesized that the sleep-push specialists would have increased stability, evidenced by lower CoM height and that their muscle activity would show greater reproducibility across trials, relative to non-specialists. We also posited that EMG power may evolve differently between our two groups suggesting that specialists will demonstrate higher reproducibility in muscular activity as previously proposed in elite versus novice athletes (Cheron et al., 2011). Potential differences may be used to create more effective coaching or training strategies for the field hockey community.

## Methods

### Subjects and experimental conditions

Data were collected from 10 hockey players of the Belgian national team (Red Lions, Olympic Gold Medalists, 2020). Players were in good health, agreed to take part in this study, and signed an informed consent explaining the purpose of the study. All the recordings were performed in a single session during the off-season. Approved by the Ethic comity of CHU Brugmann, and were conducted in conformity with the European Union directive 2001/20/EC of the European Parliament.

5 subjects were specialists in the sleep push and 5 non-specialists. Each participant completed 10 trials, each corresponding to one sleep-push movement. Players began with their back to a standard field hockey goal, turned 180°, paused in a start position, approached the ball, and completed the shot.

### Data acquisition and processing

**Figure 1:**
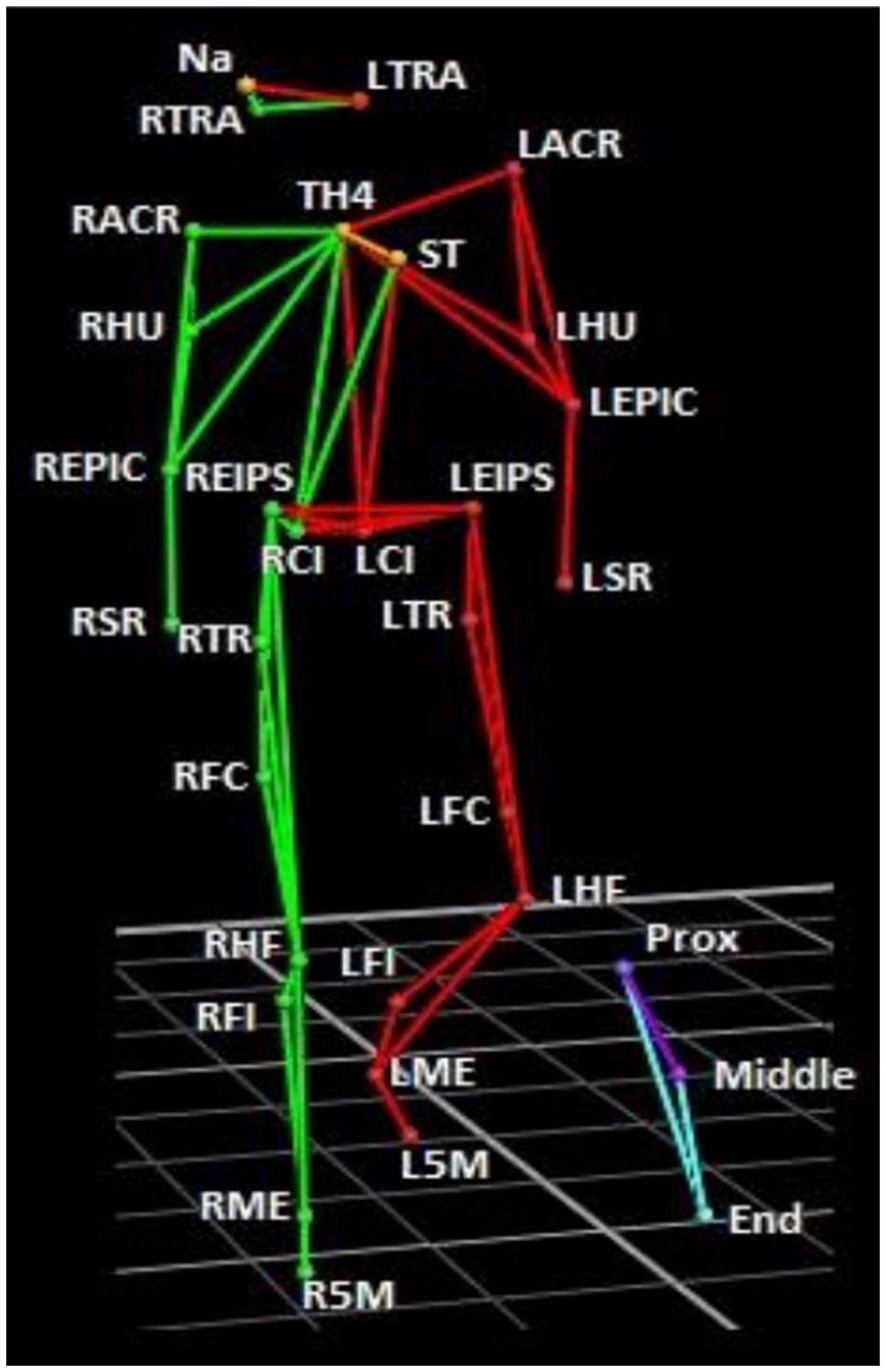
Marker set-up. Green segments correspond to the right side, and red, the left side. The stick is shown in blue/purple. Markers were placed on the skin at the following anatomical positions: Nasion (NA), right tragus (RTRA), left tragus (LTRA), sternum (ST), 4th thoracic vertebrae (TH4), left and right acromion (LACR, RACR), humerus (HU), epicondyle (EPIC), styloid process of radius (SR), anterior superior iliac spine (EIPS), iliac crest (IC). 6 left and right greater trochanter (LTR, RTR), left and right lateral femoral condyle (LFC, RFC), left and right head of the fibula (LHF, RHF), left and right fibula (LFI, RFI), left and right lateral malleolus (LME, RME), and left and right 5th metatarsal (L5M, R5M). 3 markers were also attached to the stick: the first situated closest to the hand (Prox), the next in the middle (Middle), and the third at the distal end (End) of the stick.

Whole-body kinematics were recorded using a Vicon motion capture system (Vicon, Oxford, United Kingdom). The 10-camera system detects retroreflective markers at a sampling rate of 200 Hz. The cameras were positioned around the subject to record player movements. 29 markers were attached to each subject (using Vicon Bio Mind asymmetric model; Figure 1). 3 markers were positioned on the head: nasion (NA), right tragus (RTRA), and left tragus (LTRA). 2 markers on the upper body: sternum (ST) and 4^th^ thoracic vertebrae (TH4). 4 markers were positioned on both the left (L) and right (R) arms: acromion (ACR), humerus (HU), epicondyle (EPIC) and styloid process of radius (SR). 2 markers on the pelvis: anterior superior iliac spine (EIPS) and iliac crest (IC). 6 markers on both legs: greater trochanter (TR), lateral femoral condyle (FC), head of the fibula (HF), fibula (FI), lateral malleolus (ME), and 5th metatarsal (5 M). 3 markers were also attached to the stick: the first situated closest to the hand (Prox), the next in the middle (Middle), and the third at the distal end (End) of the stick. The coordinate system was defined as: the *y*-axis was the direction where the subject ran and shot to the goal. The *x*-axis was perpendicular to the y-axis (horizontal ground plane), and the *z*-axis was the norm to *x*-axis and *y*-axis (corresponding to the vertical direction).

Surface electromyographic activity (EMG) was recorded with the wireless BTS Freeemg system (BTS Bioengineering, Milano, Italy) and synchronized with Vicon. Silver-silver chloride electrode pairs were placed on the following muscles of each subject using a symmetric model: wrist extensor (WE), wrist flexor (WF), lumbar extensor (LE), external oblique (EO), gluteus medius (GM), adductor longus (AL), semitendinosus (ST) and vastus lateralis (VL). EMG activity was recorded at 1000 Hz, bandpass filtered (20–450 Hz), full-wave rectified, smoothed using an averaging filter with a window size of 20 ms, and then normalized between 0 and 1. Data were extracted using *Easymove* (HumanWaves, Bruxelles, BE) and analyzed in Matlab (R2015b, MathWorks, Inc., Natick, MA). EMG recording issues led to incomplete data for one subject, therefore to keep the groups equal, EMG data was assessed in 8 subjects. In these 8 subjects, two trials (one in each group) contained recording errors, thus 78 trials were analyzed.

### Reconstruction of missing markers

Frame-by-frame analysis of trials was conducted to manually identify missed markers during the sleep-push movement. Reconstruction of the rigid body was carried out by correcting missing marker trajectories with the spline function option in Vicon. The suitability of the reconstructed bodies was visually checked and the correction was applied to ensure that no errors in rigid body movement across the sleep-push persisted.

### Shoot time and decomposition of the trial

**Figure 2:**
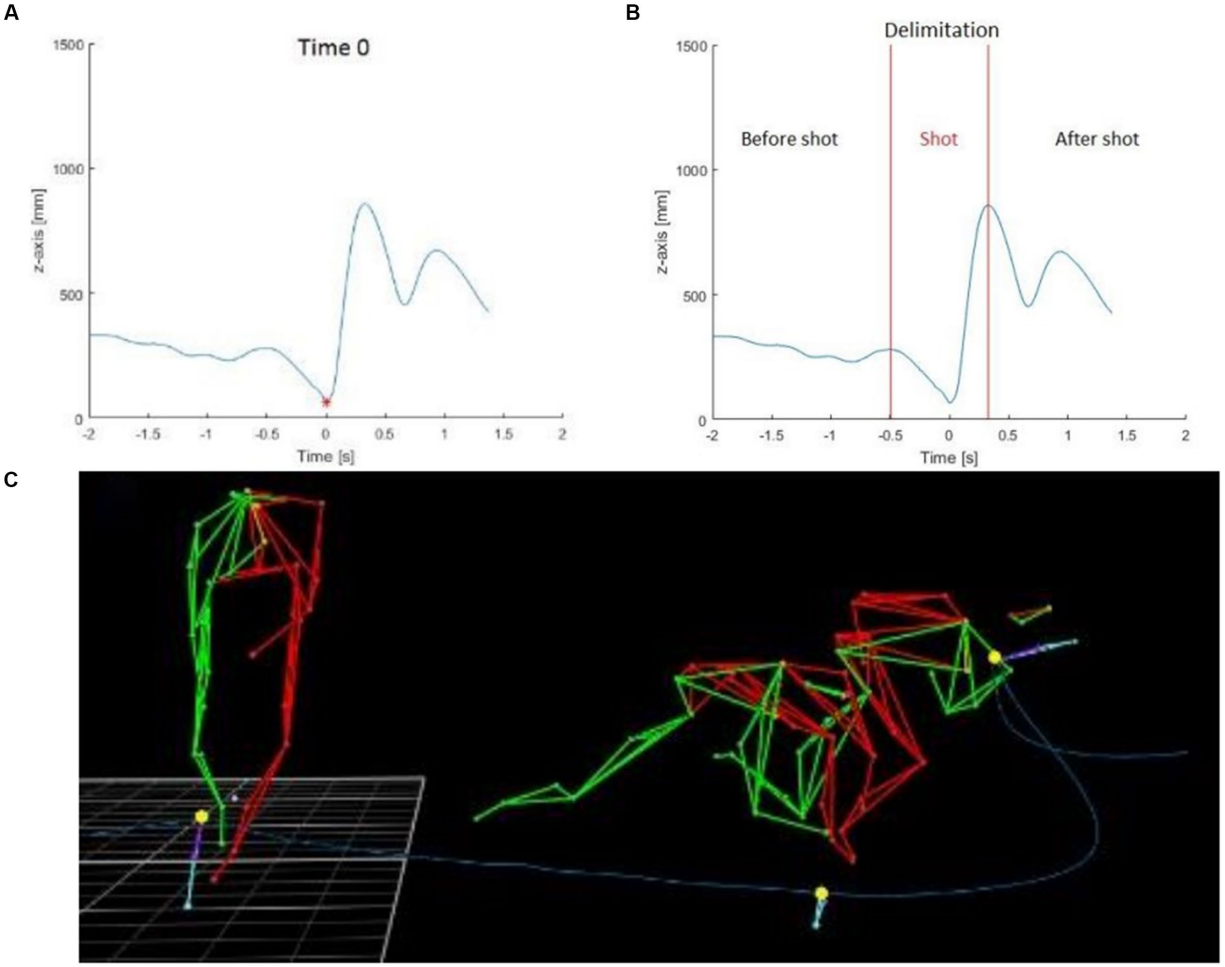
Stick height and depiction of sleep-push movement. The blue line represents the height of the Prox marker on the stick of one subject during one trial and the red point was the minimum of the Prox marker and was defined as the time 0 used to synchronize all subjects and trials between them **(A)**. The shot part was delimited between the two local maximums around the time 0 **(B)**. The three yellow points were used to delimit the shot part: start of shot part, time 0, end of the shot part **(C)**.

The sleep-push movement was broken down into three temporally distinct segments. For each trial, time 0 corresponded to the moment that the Prox marker of the stick was at its minimum height (Figure 2A). Since the pattern of the Prox marker around the time 0 followed the same path among the subjects, the shot phase was defined as the area between the two local maximums around the time 0 (Figure 2B). Furthermore, the shot phase had two distinct phases – the initial shot phase, before time 0, and the latter shot phase, after time 0. Then, the two other components of each trial included the approach phase (phase before the shot) and the follow-through phase (the phase after the shot). During the approach phase, the player moved toward the ball and transitioned into the shot phase, where the player made contact with the ball (occurs on the right side of the body), and then finished the movement with the follow-through of the stick toward their left side.

### Spatio-temporal parameters

For each subject and each trial, the times, the height of the stick, and the stick velocity were calculated for the shot phase, before time 0, and after. In addition, the Center of Mass (CoM) was calculated and reported through the variation of height and velocity.

The CoM of each subject was defined as the centroid of a 13-segment model (3 leg segments, pelvis, thorax, 2 arm segments, and head). The lower body model used in this study was based on Davis et al. (1991) and the upper body model was based on Gutierrez et al. (2003). The CoM was calculated in the x-axis based on the following formula:


$$X_{CoM}=\frac{1}{M}\overset{n}{\underset{i=1}{\sum }}x_{i}m_{i}$$

where *X_CoM_* was the position of the CoM on the *x*-axis, *m_i_* was the mass of each segment, *z_i_* was the center’s position of the segment and *M* is the body mass.

Based on the same formula, the CoM was calculated on the *y*-axis and *z*-axis.

Because of the incomplete reconstruction of CoM related to missing markers, 93 trials were calculated (45 movements made by specialists and 48 movements made by no specialists). For consistency, the same 93 trials were used to analyze all spatiotemporal parameters.

### Cross-correlation analysis

To compare the repeatability of the EMG pattern for the same muscle (auto cross-correlation) and between muscles through each trial, the normalized cross-correlation function (CFF) was calculated. The cross-correlation coefficient represents the correlation (similarity) between two series by adding a different amount of time lag (τ) between these two series. As previously described in Bengoetxea et al. (2010), the CCF function is defined as:


$$CCFd_{1}d_{2}\left(\tau \right)=\frac{1}{T\sigma_{1}\sigma_{2}}\overset{T}{\underset{0}{\int }}\left[d_{1}\left(t\right)-\mu_{1}\right]\left[d_{2}\left(t+\tau \right)-\mu_{2}\right]dt$$

where *d*_1_ and *d*_2_ were two EMG signals, μ_n,_ and σ_n_ were the mean and the variance of the signals *d*_n_. τ was the lag time between the two signals and was expressed in ms. *T* represented the time window where the CCF was performed. The calculation of the CCF has expressed a value between −1 and 1. A value of 1 corresponds to perfectly correlated signals (activation of the two muscles was performed at the same time) and a value of −1 corresponds to completely out-of-phase signals (when a muscle is activated, the other is inactivated).

CCF calculations were also used to assess different muscle interactions between specialists and non-specialists. Four functional muscle groups were identified for CCF calculation. The upper extremity muscles included: RWE, RWF, LWE, and LWF, and postural muscles: RLE, REO, LLE, and LEO. The third was the zone of the upper thigh: RGM, RAL, LGM, and LAL. And the fourth was the area of the middle thigh: RST, RVL, LST, and LVL.

### Frequency analysis

The time course of muscle activation and spectral characteristics of the EMG signals were quantified using frequency analysis. Grand average signals from each EMG were separated between the two groups for each muscle and side and compared using a *t*-test. All calculations were done with EEGLAB (Delorme and Makeig, 2004 and Matlab toolbox)

### Statistics

Descriptive statistics included means and standard deviation (SD). Since the spatiotemporal results followed non-normal distribution, Wilcoxon rank-sum tests were conducted on Matlab to detect differences between groups. The significance level was set *a priori* to 5%. To look for differences between the two groups in CoM and cross-correlation coefficient, a repeated measure ANOVA was done with Matlab. To evaluate the difference in group average and frequency analysis, a t-test was performed using EEGLAB toolbox.

## Results

### Spatiotemporal parameters

**Figure 3:**
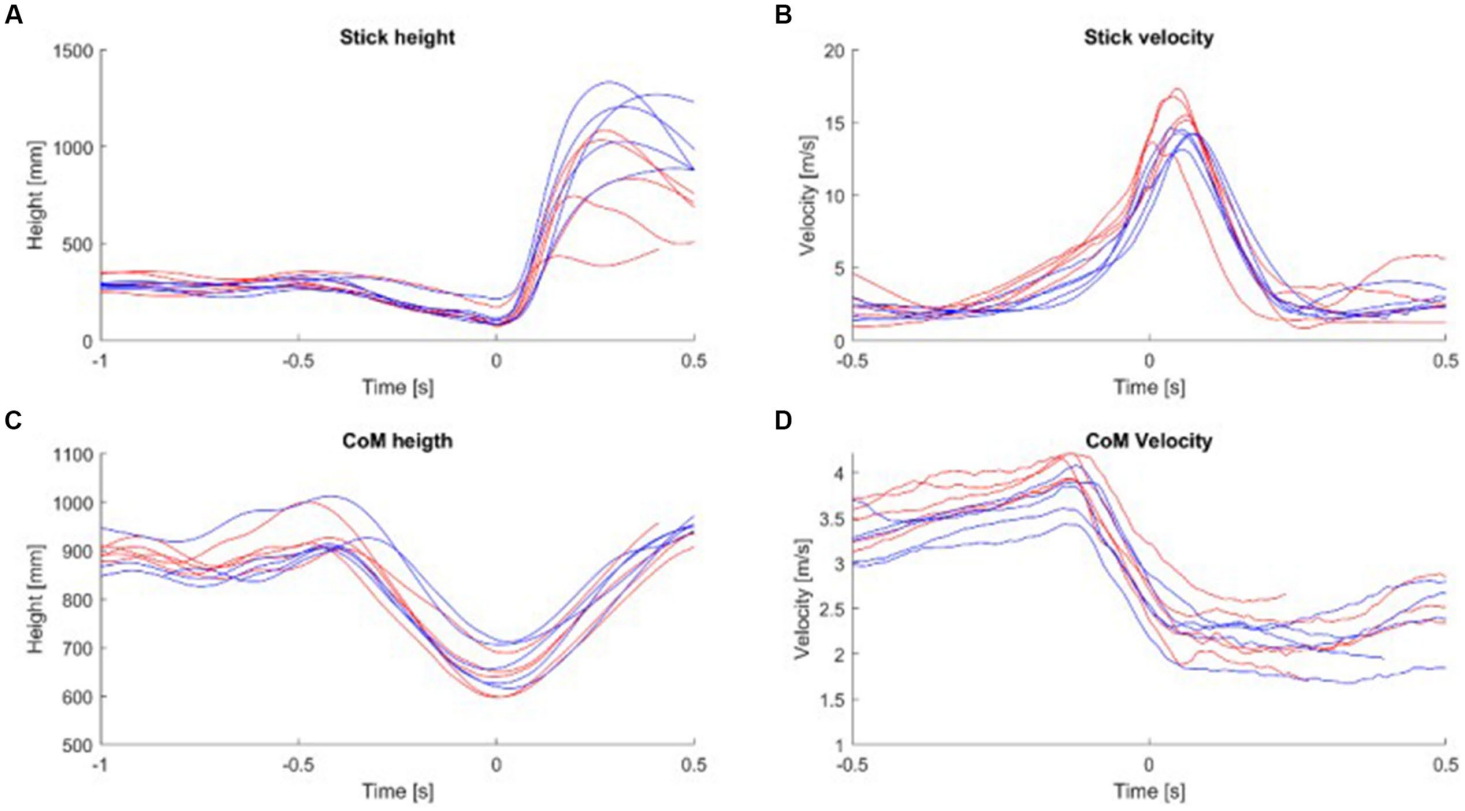
**(A)** The mean stick height was calculated for each subject. **(B)** The mean stick velocity was calculated for each subject. **(C)** The mean height of the CoM was calculated for each subject. **(D)** The mean velocity of CoM was calculated for each subject during the shot part. Red line, specialist; Blue line, non-specialist; CoM, Center of Mass.

Results for the spatiotemporal parameters are shown in Figure 3 and Table 1. The time during the initial shot phase tended to be faster for the non-specialists in comparison to the specialists (0.45 s and 0.53 s respectively), but slower during the second part (0.35 s and 0.25 s), with only this later shot duration reaching statistical difference (Table 1). These results highlighted the fact that the specialists finished the sleep push with a faster stick movement. The height of the stick was not significantly different during the initial shot phase but presented a mean value significantly lower in the specialists during the latter shot phase (Figure 3A). The stick velocity supported this finding, with a mean value for the latter shot phase of 9.02 m/s for specialists in comparison to 7.03 m/s for the non-specialists (Figure 3B). Statistically significant differences were also observed between specialists and non-specialists for the CoM: Specialists put their CoM closer to the ground during the initial shot phase and kept their CoM low during the latter shot phase (Figure 3C). They also had a higher CoM velocity during the shot and had a better ability to stabilize the movement of their body when they shot the ball, which was characterized by a higher difference in their CoM velocity during the shot (Figure 3D).

**Table 1:** Differences between specialist and non-specialists of the sleep-push for several spatio-temporal parameters during different parts of the shot.

Spatio-temporal parameters	Area of interest	Non-specialists	Specialists
Duration of the shot [s]	Full shot	0.81 ± 0.10	0.77 ± 0.09
	First part of the shot	0.45 ± 0.06	0.53 ± 0.05
	Second part of the shot*	0.35 ± 0.07	0.24 ± 0.06
Stick velocity [m/s]	Full shot*	4.94 ± 0.36	5.74 ± 0.21
	First part of the shot*	3.45 ± 0.35	4.31 ± 0.41
	Second part of the shot*	6.98 ± 0.97	9.17 ± 1.28
Stick height [mm]	Full shot*	438 ± 69	307 ± 29
	First part of the shot	212 ± 32	222 ± 35
	Second part of the shot*	722 ± 112	479 ± 120
Variation CoM height [mm]	Full shot	277 ± 47	297 ± 63
	First part of the shot	270 ± 43	228 ± 62
	Second part of the shot*	296 ± 64	141 ± 52
CoM velocity[m/s]	Full shot*	2.89 ± 0.23	3.23 ± 0.24
	First part of the shot	3.4 ± 0.25	3.64 ± 0.2
	Second part of the shot	2.22 ± 0.27	2.31 ± 0.33
Variation CoM velocity [m/s]	Full shot	1.91 ± 0.22	2.11 ± 0.32
	First part of the shot	1.28 ± 0.22	1.46 ± 0.28
	Second part of the shot	0.68 ± 0.22	0.71 ± 0.25

The values in the table are indicated as mean ± standard deviation. The variation of CoM height was defined as the difference between the maximum and the minimum of CoM height during the aera of interest. The variation in CoM velocity was defined as the difference between the maximum and the minimum of CoM velocity during the area of interest.

CoM, Center of mass.

*Statistically significant differences among specialists and non-specialists (*p* ≤ 0.05).

### Mean EMG activity during the sleep-push movement

**Figure 4:**
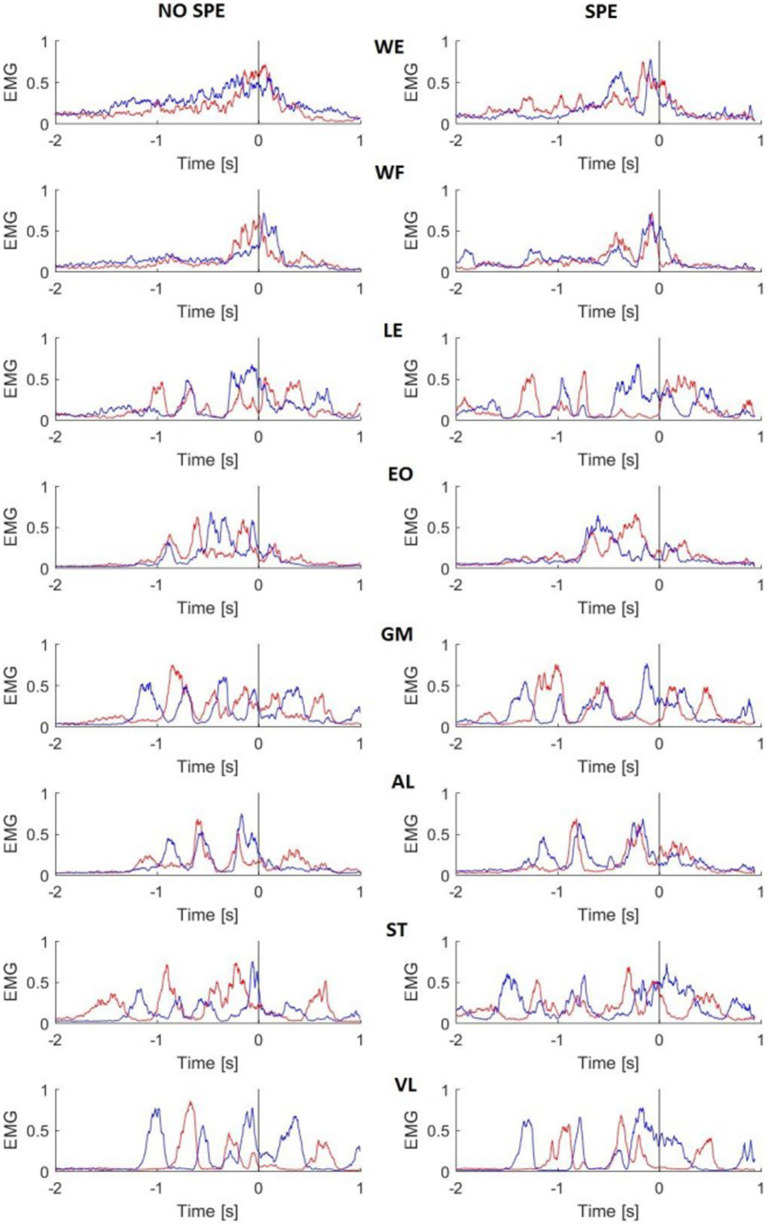
Mean EMG activity of two representative subjects. Red lines are the right side of the body, blue lines are the left side. The black line represents the time 0. EMG activity was rectified, smoothed, and normalized for each trial. NO SPE, non-specialist; SPE, specialist; WE, wrist extensor; WF, wrist flexor; LE, lumbar extensor; EO, external oblique; GM, gluteus medius; AL, adductor longus; ST, semitendinosus; VL, vastus lateralis.

Figure 4 illustrates the 8 superimposed muscle activation patterns recorded on the left (blue line) and right (red line) sides of the body, 2 s before and 1 s after the shot in a non-specialist and specialist representative subjects. The sleep push was characterized by complex combinations of EMG patterns. For all recorded muscles except for the WE and WF, the activation patterns presented more than 2 bursts of muscle activity. For the WE, the non-specialist showed a ramp-like activation of both arms while the specialist presented more bursting activity before the shot. The same distinction was also present in the WF activation. It is interesting to note that the reciprocal left and right bursting activity of the ST muscle both subjects. The VL possessed similar reciprocal bursting patterns. Other muscles, best evidenced by AL, showed synchronous left and right muscle activity before the shot.

### Auto cross-correlation to assess the repeatability of activation

**Figure 5:**
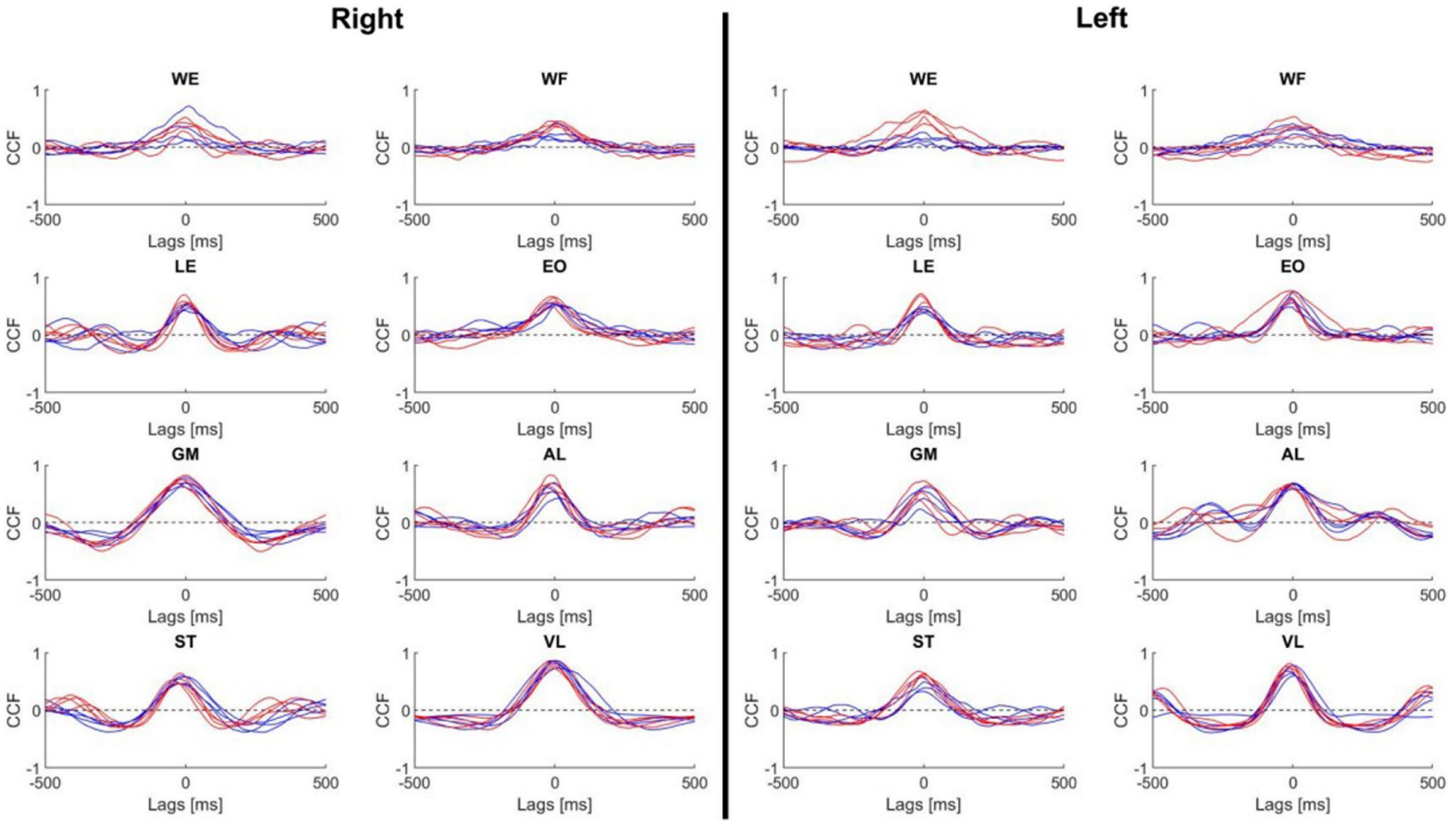
Mean of the auto cross-correlation curves for each muscle and each subject. Cross-correlation function was performed during the shot with a time window of 500 ms. Red line, specialist; Blue line, non-specialist. WE, wrist extensor; WF, wrist flexor; LE, lumbar extensor; EO, external oblique; GM, gluteus medius; AL, adductor longus; ST, semitendinosus; VL, vastus lateralis; CCF, cross-correlation function.

To show if the specialist’s muscles were more similar during the repeated movement, auto CCF were performed for all the trials and each muscle. The superimposition of the auto CCF demonstrated a high degree of reproducibility of each muscle and both body sides across players (Figure 5). Only the WE muscle of the left side presented a greater CCF max in the specialists. Similarly, the left ST muscle demonstrated a similar pattern, although to a lesser extent (Table 2). The time lags remained close to zero except for the WE of the left side presenting a larger and highly variable time lag in the non-specialist group (−67 ms versus −2.7 ms for the specialists). The reverse situation occurred for the ST muscle of the right side presenting a larger time lag with less variability for the specialists.

**Table 2:** Auto cross-correlation coefficients and time lags for the reproduction of the sleep-push.

	Right				Left			
	Non-spe		Spe		Non-spe		Spe	
	CCF	Lag (ms)	CCF	Lag (ms)	CCF	Lag (ms)	CCF	Lag (ms)
WE	0.50 ± 0.09	3.73 ± 131.19	0.50 ± 0.10	−1.32 ± 84.14	**0.36 ± 0.11**	**−67.13 ± 189.81**	**0.63 ± 0.08**	**−2.79 ± 41.40**
WF	0.45 ± 0.11	12.13 ± 157.44	0.54 ± 0.10	13.81 ± 98.75	0.45 ± 0.11	23.02 ± 142.31	0.51 ± 0.08	2.28 ± 73.09
LE	0.66 ± 0.09	2.21 ± 63.748	0.73 ± 0.07	5.43 ± 27.14	0.64 ± 0.09	−2.37 ± 54.75	0.72 ± 0.08	−4.70 ± 30.66
EO	0.67 ± 0.11	11.52 ± 75.35	0.66 ± 0.09	−9.80 ± 28.46	0.74 ± 0.10	−10.99 ± 54.12	0.75 ± 0.07	−10.99 ± 25.63
GM	0.80 ± 0.05	−0.18 ± 40.76	0.82 ± 0.05	−12.54 ± 29.29	0.67 ± 0.09	7.99 ± 70.76	0.69 ± 0.08	−11.30 ± 80.70
AL	0.74 ± 0.08	12.81 ± 55.99	0.79 ± 0.05	−12.63 ± 29.60	0.76 ± 0.06	3.15 ± 45.66	0.75 ± 0.07	−6.30 ± 33.31
ST	0.66 ± 0.09	**−2.33 ± 60.58**	0.70 ± 0.07	**−23.75 ± 37.68**	**0.62 ± 0.09**	−0.85 ± 75.96	**0.71 ± 0.08**	−4.16 ± 28.62
VL	0.88 ± 0.04	5.04 ± 29.13	0.89 ± 0.04	−4.54 ± 27.16	0.84 ± 0.06	−8.47 ± 50.43	0.86 ± 0.05	−9.84 ± 22.44

Cross-correlation function was performed during the shot with a time window of 500 ms.

Values are means ± standard deviation.

CCF, Cross-correlation function; WE, wrist extensor; WF, wrist flexor; LE, lumbar extensor; EO, external oblique; GM, gluteus medius; AL, adductor longus; ST, semitendinosus; VL, vastus lateralis.

Bold values indicate significant differences between non-specialists and specialists (*p* ≤ 0.05).

### Cross-correlation between muscles

**Figure 6:**
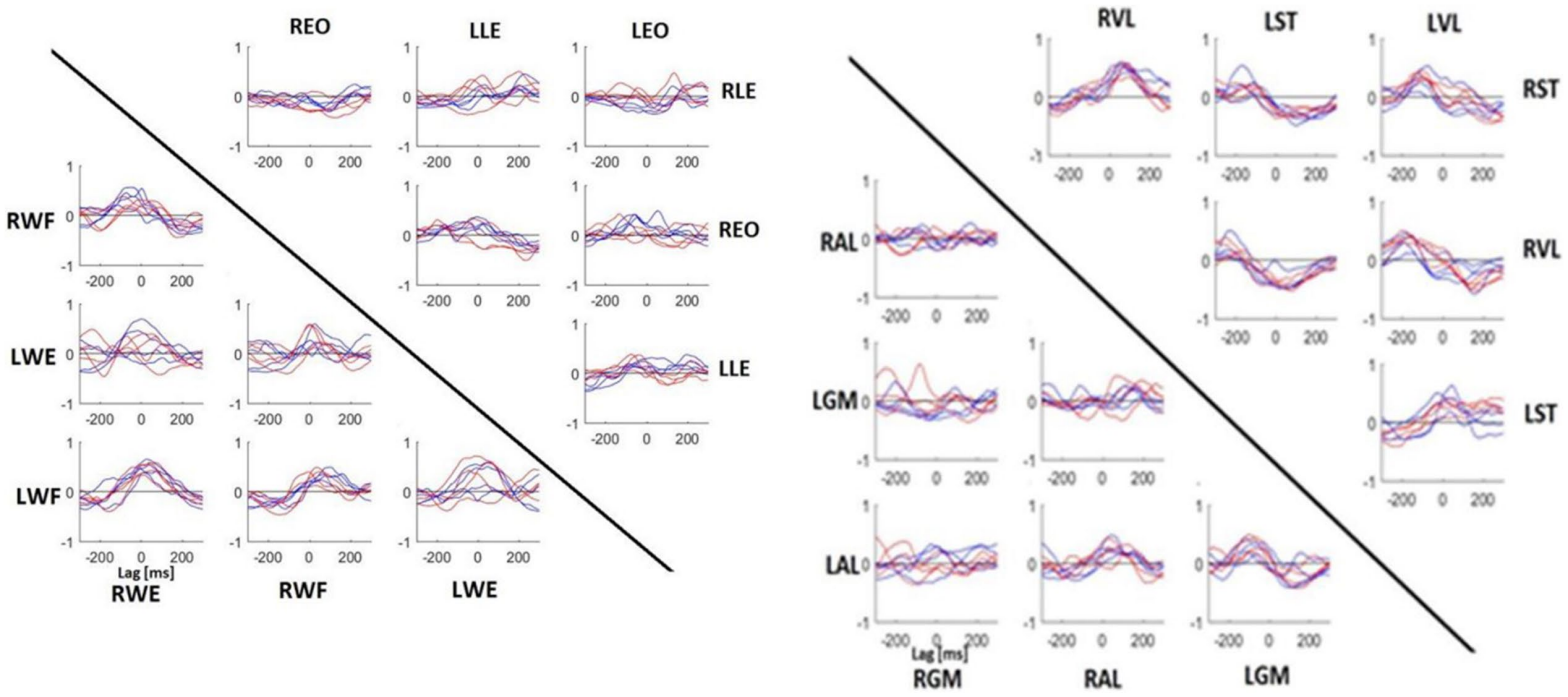
Mean CCF between muscles of upper (left) and lower (right) body parts. Each panel indicates the CCF between muscles represented on the horizontal line and vertical line. Red line, specialists. Blue line, no-specialists. If the peak value was on the left of lag 0, it meant that the muscle written on the horizontal line was activated before the muscle written on the vertical line. If the peak value was on the right, it was the inverse. WE, wrist extensor; WF, wrist flexor; LE, lumbar extensor; EO, external oblique; GM, gluteus medius; AL, adductor longus; ST, semitendinosus; VL, vastus lateralis.

Figure 6 illustrates the CCF performed between the muscles of the upper and lower parts of the body. It was not possible to make a clear distinction between specialist and non-specialist athletes using CCF analysis. However, in the comparison of these CCF pairs, we may highlight the following characteristics of muscle coordination during the shot: (1) the CCF between LWF and RWE were synchronized, (2) the RWF was activated before RWE, (3) the postural muscle (LLE, LEO, REO and RLE) did not present a clear CCF peak, (4) the LAL muscle was activated 100 ms before the LGM muscle, (5) the RST was activated 100 ms before the LVL and 100 ms after the RVL; (6) LST activity shared similar timecoures with RST and RVL activity.

### Frequency analysis

**Figure 7:**
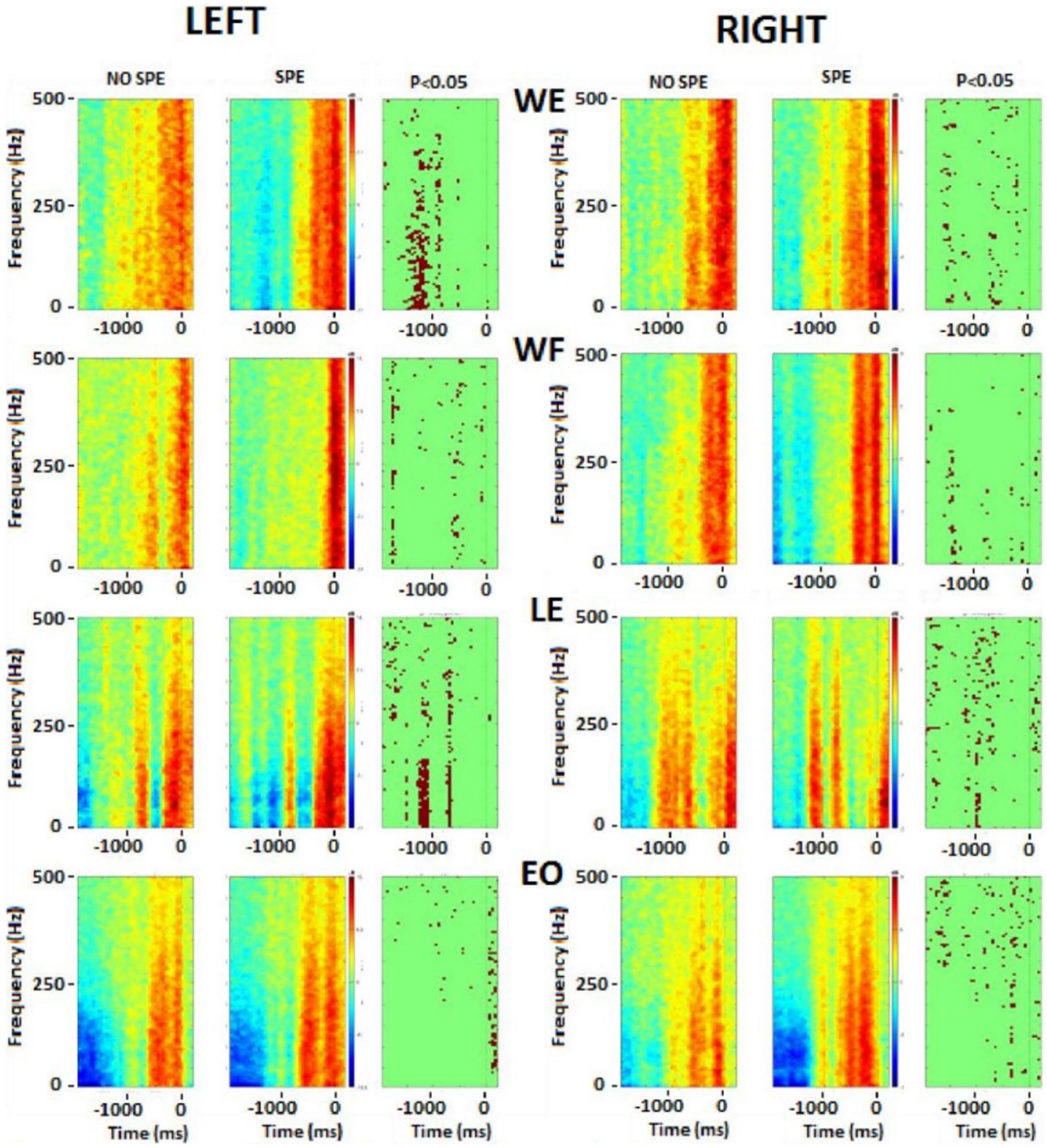
Frequency analysis performed on the upper body muscles. Left column represents the left muscles and right column represents right muscles. In each picture, the first square is the result for non-specialists, the second square is the result for specialists and the last one is the t-test result. Red color, higher power, blue colour, lower power. The singular points (third column on each side) represent significant differences between groups. WE, wrist extensor; WF, wrist flexor; LE, lumbar extensor; EO, external oblique.

To compare the results more specifically, an average for each group was done during −2 s to 0.31 s (the shortest EMG time of all subjects) and a t-test was performed. Figure 7 illustrates the *t*-test results. The difference between the two groups started with the upper body at the beginning of the movement and then went down to the lower body when doing the shot.

**Figure 8:**
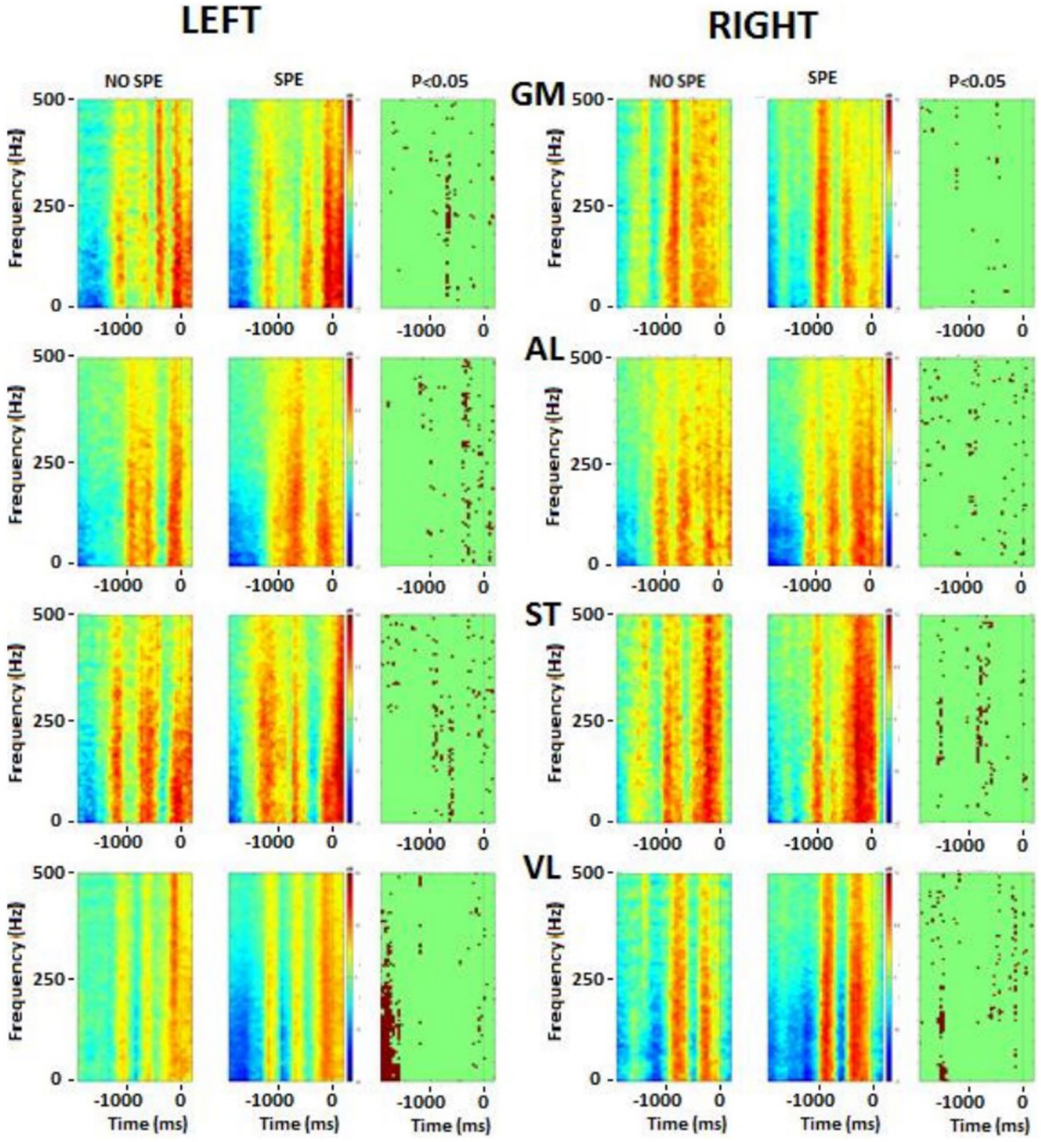
Frequency analysis performed on the lower body muscles. Left column represents the left muscles and right column represents right muscles. In each picture, the first square is the result for no-specialists, the second square is the result for specialists and the last one is the *t*-test result. Red colour, higher power, blue colour, lower power. The singular points (third column on each side) represent significant differences between groups. GM, gluteus medius; AL, adductor longus; ST, semitendinosus; VL, vastus lateralis.

There were significant differences in the flexor and extensor muscles of the wrist before the shot, but during the shot, the activation tended to remain the same. Immediately before starting the shot, differences occurred in the postural muscles (RLE, REO, and LLE). During the shot, there were significant differences in the leg muscles (RAL, RST, RVL, LGM, and LAL). As explained above, the frequency of each EMG was calculated and then averaged by groups. These results were plotted on a color image representing the frequency power of upper and postural (Figure 7) and lower (Figure 8) body muscles. The left wrist muscle (WE) of the specialist group presented a lower power before and −1 s and a short increase of power at 0 while the non-specialist maintained a power increase during the preparatory period and a lower power at the time of the shot. The same muscle strategy in favor of EMG inhibition was recorded in other upper limb muscles (right WE, left and right WF, and left LE) of the specialist group (Figure 7). The same tendency of short EMG inhibition in the specialist group was also found for some muscles of the lower body such as the right ST and the left and right VL (Figure 8).

The lumbar extensor muscles of specialists were more synchronized between the right side and the left side. For the right external oblique, specialists seemed to activate this muscle longer compared to non-specializists before the shot, while the left side was seemed to be more active during and immediately after the shot. The gluteus medius (GM) had the same pattern between groups for the right side (no statistical difference), while the left side was different before the shot part. In the specialist, one activation was visible around −0.5 ms while for the non-specialist group, two activations were presented. There was also a slight difference after the time 0: the specialists activated the muscle more strongly.

The right adductor longus (AL), possessed bursting activity in across both groups, with each burst occuring before the shot (− 0.5 s) and lasting approximately 500 ms. On the left side, burst behavior looked similar in both groups, with specialists tending to have longer and more intense contractions.

For the two groups, the ST muscle showed reciprocal activation patterns between the right and the left side, meaning with the left was active, the right was not, and vice versa. The VL showed the same pattern between groups, although the muscles from both sides were less activated for specialists at the beginning of the movement, and the right side had a longer activation time just before the time 0.

Three main results were thus extracted from these figures: (1) specialists’ muscle showed less activity during the approach phase, (2) specialists seemed to possess stronger muscle activity with more precise bursting patterns, and (3) burst duration in the lower body was similar between groups and tended to last approximately 500 ms.

## Discussion

The present study showed that compared to non-specialists, sleep-push shot specialist players had significantly increased stick velocity during the second part of the shot. Specialists also showed a significantly lower power spectrum in the activity of the upper limb muscles before the shot. Superimposition of the auto crosscorrelation demonstrates a high degree of reproducibility in muscle activations. The sleep-push shot in field hockey is a dynamic movement, requiring the coordinated effort of the entire body. Across sports science literature, biomechanical and muscle-based properties are typically compared between those with specific sports experience and their untrained counterparts. Less common is the comparison of elite athletes within the same discipline; however, this type of study design may be more informative for developing coaching or training strategies for elite-level athletes. Indeed, our analysis highlights distinct kinematic and muscle activation strategies depending on player specialization. Our results indicate that specialists tend to finish their sleep-push shot with a lower CoM, reach a higher stick velocity, and possess stronger muscle inactivation before the shot phase of the sleep-push. CCF analysis revealed similar muscle activation reproducibility across both groups. One of the main limitations of this study is the fact that the two groups were uniquely categorized with respect to their ability to perform the sleep-push movement. It would have been very interesting to have access to additional information such as anthropometric data, years of practice in the discipline, and the details of the specific training followed by the specialist players in the sleep-push movement.

Field hockey coaches may thus take advantage of these findings to measure the progress of players or evaluate the effectiveness of a training paradigm. Coaching with an emphasis on biomechanical principles (Knudson, 2007) may improve performance. In contrast, attentional focus on muscles during exercise resulted in poor muscle coordination (Lohse and Sherwood 2012). Nonetheless, empirical data from the sleep-push movement reveals the training-specific differences across players – the question remains as to how this information can be translated into meaningful coaching strategy.

### Stick movement and CoM height

Because the goal of the sleep-push is to shoot a ball past a goalie and/or defenders into a fixed space, it would follow that a higher speed shot may be more advantageous because it is more difficult to defend. We show that stick velocity was higher for the specialized compared to the non-specialized players (Table 1). This result supports previous studies showing that experts possessed increased velocity when performing a shot or explosive actions in sport (van den Tillaar and Ettema, 2006; Rousanoglou et al., 2010; Maffiuletti et al., 2016; Del Vecchio et al., 2019; Mota et al., 2019). Measurement of stick velocity may be a useful tool for evaluating the effectiveness of sleep-push training in a highly competitive sporting environment. Though our experimental set-up used a motion capture system to assess stick velocity, it may be more accessible to use an accelerometer to measure velocity in non-laboratory settings.

Another important feature of sport, especially during and after complicated movements, is postural stability. The sleep-push movement is characterized by the dynamic contortion of the upper extremities relative to the lower body, and without proper balance, shot success may suffer. We report that specialists possessed a lower CoM as they finished their shot, compared to the non-specialists (Figure 3 and Table 1). Importantly, in the context of a field hockey game, after completing a sleep-push movement, i.e., taking a shot on goal, the player must react and move according to the shot outcome. This means that ending the shot in a more stable position may encourage a more efficient reactive movement after the shot, whereas a less stable position may add precious time because corrective movements may be necessary. Some studies have demonstrated improved stabilization in experts of a specific movement compared to novices (Era et al., 1996; Davlin, 2004). Furthermore, anticipatory postural adjustments (APAs), important for stability maintenance during dynamic movements, have been shown to modulate with training. Motor performance appears to benefit from these changes in training-related improvement of APAs (Saito et al., 2014; Cavallari et al., 2016). In this view, specialized training promotes an increased ability to correct postural disturbances, with low CoM height representing a tangible metric for postural evaluation.

### Auto and cross-correlation within muscles and between muscles

The present results indicate a predominance of muscular inhibition in the sleep push specialists which might be in line with a ballistic mode of control implying a pre-movement inhibition before the acting pulse and clearcut reciprocal inhibition between the antagonistic muscle couples. Interestingly, it has been shown that long-term training modulates the cortical inhibitory command in a task- and muscle-specific manner where explosive training is followed by decreased intracortical inhibition and improvement of the associated performance (Taube et al., 2020).

Muscle activation reproducibility, as evaluated by the CCF coefficient, revealed similarities between the two groups. Our auto cross-correlation analysis showed that both athlete groups had high reproducibility of muscles between trials, with player specialization corresponding to three significantly different CCF coefficients: left wrist flexor, right external oblique, and right gluteus medius. Although these three muscles possessed increased mean CCF coefficients, to argue that muscle activation reproducibility is enhanced in the specialized players would be inaccurate. It is important to remember that all athletes in this study are elite, meaning they are top performers in their sport at the country level. Due to their generalized training over time, it can be speculated that persistent stick practice, whether shooting or passing, promotes patterned muscle activation. Without consistent and reliable muscle activation, field hockey play may become difficult. Therefore, the similar reproducibility of muscle activation between trials for both specialized and non-specialized players is in line with idea that elite play may be dependent on enhanced muscle coordination, which is not necessarily unique to a singular movement type.

In accordance with the auto cross-correlation, muscle comparisons of muscle activity via cross-correlation revealed similarities across the specialists and non-specialists. No consistent findings were identified, demonstrating that the muscle activation patterns in the sleep-push movement were quite similar between groups. The lack of difference may be due to a similar time-course of muscle activation or due to high variability among the participants. This may be improved in the future by increasing the sample size. Importantly, variability could be attributed to noise in the measurement caused by dynamic movement (i.e., movement artifact), or differing muscle activity strategies that could reflect varying mechanical approaches for the same movement.

### Frequency analysis

Analysis of the sleep-push movement in the frequency domain allowed for the assessment of EMG spectral power over time. The evolution of muscle activity across muscles and groups remained relatively consistent over the sleep-push movement. Upper extremity and lower extremity muscles, when active, tended to possess similar, although not identical frequency components (see Figures 7, 8). This indicates that active muscles were operating at similar frequencies across the movement and this is evidenced in the sparseness of points in the right columns of the respective figures, where points represent significant differences between groups. However, the left wrist extensor muscle and lumbar extensor muscle revealed a dense collection of points indicating significant differences between groups. Compared to the non-specialists, specialists of the sleep-push had significantly less active muscles at time − 1,000 ms. This finding indicates a relaxation during the sleep-push movement, potentially giving rise to a more efficient movement.

Similarly, the lower extremity possessed little difference between groups. The left vastus lateralis possessed the clearest difference between groups (see Figure 8), where specialists had less activation ~1,200 ms before time 0. Again, this muscle coordination strategy may enable more fluid movement as the shot approach requires dynamic knee flexion, and vastus lateralis relaxation may serve to promote a more effective sleep-push strategy. Importantly, the most notable differences in the frequency plots occur on the left side and demonstrate inactivation of a muscle in the specialist players. This finding may be useful for coaches and players, where awareness of muscle-based cues could be used during sleep-push practice.

## Conclusion

The ability to detect kinematic and muscle-based differences across a performance spectrum would confirm that specialized movement training is effective. Indeed, we have identified unique features of the sleep-push movement in players specializing in the sleep-push movement. Our most salient findings include increased stick velocity during the shot, low CoM height after the shot, and increased muscle relaxation upon shot approach.

As expected, the players specializing in the sleep-push movement possessed different traits than their non-specialized counterparts. These differences, within an elite group of athletes, highlight factors that may be exploited by coaching and/or training strategy. As reported across a range of sporting disciplines, motor learning may be enhanced in athletes, compared to non-athletes (di Cagno et al., 2014; Verburgh et al., 2016; Seidel et al., 2017). Elite athletes may stand to benefit from studies of their elite teammates or competition, where they can easily integrate findings into their performance strategy. Additionally, with the growth of sports science within the professional sports community, studies like this one are becoming more feasible. Accessibility of instrumentation, ability to analyze and interpret data, and subsequently translate into a real sports setting are the pivotal steps that must be taken for athletes to benefit.

## Data availability statement

The data analyzed in this study is subject to the following licenses/restrictions: the raw data supporting the conclusions of this article will be made available by the authors, without undue reservation. Requests to access these datasets should be directed to Ana.Maria.Cebolla.Alvarez@ulb.be.

## Ethics statement

This study was reviewed and approved by Ethics committee of CHU Brugmann (Belgium) and conducted in conformity with the European Union directive 2001/20EC of the European Parliament. The patients/participants provided their written informed consent to participate in this study.

## Author contributions

A-MC and GC conceived the original idea. GC, MP, and A-MC designed the experiment. MP and GC performed the experiments. KC performed the data analysis. KC and GC wrote the first version of the manuscript. SM and A-MC contributed to the writing of this manuscript. All authors contributed to the article and approved the submitted version.

## Funding

This work was funded by research funds from the Université Libre de Bruxelles (ULB), Belgium, the Sports Ministry of the Federation Wallonia-Brussels, the ADEPS, the Fonds G. Leibu and the Fonds Brain Society. SM is supported by an Aspirant research fellowship awarded by the F.R.S.-FNRS (Belgium). This study was also supported by Anne-Marie Clarinval the Human Waves spin-off.

## Conflict of interest

The authors declare that the research was conducted in the absence of any commercial or financial relationships that could be construed as a potential conflict of interest.

## Publisher’s note

All claims expressed in this article are solely those of the authors and do not necessarily represent those of their affiliated organizations, or those of the publisher, the editors and the reviewers. Any product that may be evaluated in this article, or claim that may be made by its manufacturer, is not guaranteed or endorsed by the publisher.

## References

[ref1] Amblard B. Assaiante C. Lekhel H. Marchand A. R. (1994). A statistical approach to sensorimotor strategies: conjugate cross-correlations. J. Mot. Behav. 26, 103–112. doi: 10.1080/00222895.1994.9941665 15753063

[ref2] Barnamehei H. Tabatabai Ghomsheh F. Safar Cherati A. Pouladian M. (2018). Upper limb neuromuscular activities and synergies comparison between elite and nonelite athletics in badminton overhead forehand smash. Appl. Bionics Biomech. 2018:6067807. 30671132 10.1155/2018/6067807 PMC6317092

[ref3] Bengoetxea A. Dan B. Leurs F. Cebolla A. M. De Saedeleer C. Gillis P.. (2010). Rhythmic muscular activation pattern for fast figure-eight movement. Clin. Neurophysiol. 121, 754–765. doi: 10.1016/j.clinph.2009.12.021 20075001

[ref4] Cavallari P. Bolzoni F. Bruttini C. Esposti R. (2016). The organization and control of intra-limb anticipatory postural adjustments and their role in movement performance. Front. Hum. Neurosci. 10:525. doi: 10.3389/fnhum.2016.00525 PMID: 27807411 PMC5069406

[ref5] Cheron G. (2015). From biomechanics to sport psychology: the current oscillatory approach. Front. Psychol. 6:1642. doi: 10.3389/fpsyg.2015.01642 26582999 PMC4628124

[ref6] Cheron G. Bengoetxea A. Bouillot E. Lacquaniti F. Dan B. (2001). Early emergence of temporal co-ordination of lower limb segments elevation angles in human locomotion. Neurosci. Lett. 308, 123–127. doi: 10.1016/s0304-3940(01)01925-5 PMID: 11457575

[ref7] Cheron G. Bengoetxea A. Pozzo T. Bourgeois M. Draye J. P. (1997). Evidence of a preprogrammed deactivation of the hamstring muscles for triggering rapid changes of posture in humans. Electroencephalogr. Clin. Neurophysiol. 105, 58–71. doi: 10.1016/s0924-980x(96)96544-3 PMID: 9118840

[ref8] Cheron G. Duvinage M. Casterman T. Leurs F. Cebolla A. Bengoetxes A.. (2011). “Toward an integrative dynamic recurrent neural network for sensorimotor coordination dynamics” in Recurrent neural networks for temporal data processing. eds. Cardot H. Boné R. (InTech), 65–80.

[ref10] d’Avella A. Lacquaniti F. (2013). Control of reaching movements by muscle synergy combinations. Front. Comput. Neurosci. 7:42. doi: 10.3389/fncom.2013.00042 23626534 PMC3630368

[ref11] Davis R. B. Õunpuu S. Tyburski D. Gage J. R. (1991). A gait analysis data collection and reduction technique. Hum. Mov. Sci. 10, 575–587. doi: 10.1016/0167-9457(91)90046-Z

[ref12] Davlin C. D. (2004). Dynamic balance in high level athletes. Percept Mot Skills. 98, 1171–1176. doi: 10.2466/pms.98.3c.1171-1176 15291203

[ref9004] Delorme A. Makeig S. (2004). EEGLAB: an open source toolbox for analysis of singletrial EEG dynamics including independent component analysis. J Neurosci Methods. 134, 9–21. doi: 10.1016/j.jneumeth.2003.10.009 15102499

[ref13] de Subijana C. L. Juárez D. Mallo J. Navarro E. (2011). The application of biomechanics to penalty corner drag-flick training: a case study. J. Sports Sci. Med. 10, 590–595. PMID: 24150638 PMC3737828

[ref14] Del Vecchio A. Negro F. Holobar A. Casolo A. Folland J. P. Felici F.. (2019). You are as fast as your motor neurons: speed of recruitment and maximal discharge of motor neurons determine the maximal rate of force development in humans. J. Physiol. 597, 2445–2456. doi: 10.1113/JP277396, PMID: 30768687 PMC6487919

[ref15] di Cagno A. Battaglia C. Fiorilli G. Piazza M. Giombini A. Fagnani F.. (2014). Motor learning as young gymnast’s talent indicator. J. Sports Sci. Med. 13:767. 25435768 PMC4234945

[ref16] Dorel S. Couturier A. Hug F. (2008). Intra-session repeatability of lower limb muscles activation pattern during pedaling. J. Electromyogr. Kinesiol. 18, 857–865. doi: 10.1016/j.jelekin.2007.03.002 17449281

[ref17] Elferink-Gemser M. T. Visscher C. Lemmink K. A. P. M. Mulder T. (2007). Multidimensional performance characteristics and standard of performance in talented youth field hockey players: a longitudinal study. J. Sports Sci. 25, 481–489. doi: 10.1080/02640410600719945, PMID: 17365535

[ref18] Era P. Konttinen N. Mehto P. Saarela P. Lyytinen H. (1996). Postural stability and skilled performance--a study on top-level and naive rifle shooters. J. Biomech. 29, 301–306. doi: 10.1016/0021-9290(95)00066-6 PMID: 8850636

[ref19] Feldman A. G. Adamovich S. V. Levin M. F. (1995). The relationship between control, kinematic and electromyographic variables in fast single-joint movements in humans. Exp. Brain Res. 103, 440–450. doi: 10.1007/BF00241503 7789450

[ref20] Filli L. Meyer C. Killeen T. Lörincz L. Göpfert B. Linnebank M.. (2019). Probing corticospinal control during different locomotor tasks using detailed time-frequency analysis of electromyograms. Front. Neurol. 10:17. doi: 10.3389/fneur.2019.00017 30761064 PMC6361808

[ref21] Garcia C. Barela J. A. Viana A. R. Barela A. M. (2011). Influence of gymnastics training on the development of postural control. Neurosci. Lett. 492, 29–32. doi: 10.1016/j.neulet.2011.01.047, PMID: 21276829

[ref22] Gaveau V. Pisella L. Priot A.-E. Fukui T. Rossetti Y. Pélisson D.. (2014). Automatic online control of motor adjustments in reaching and grasping. Neuropsychologia 55, 25–40. doi: 10.1016/j.neuropsychologia.2013.12.005, PMID: 24334110

[ref23] Gómez M. López de Subijana C. Antonio R. Navarro E. (2012). Kinematic pattern of the drag-flick: a case study. J. Hum. Kinet. 35, 27–33. doi: 10.2478/v10078-012-0076-7, PMID: 23487429 PMC3588699

[ref24] Goods P. S. R. McKay A. K. Appleby B. Veli D. Peeling P. Jennings D. (2022). A repeated shuttle sprint test with female and male international field hockey players is reliable and associated with single sprint but not intermittent endurance performance. PLoS One 17:e0271244. doi: 10.1371/journal.pone.0271244, PMID: 35830427 PMC9278775

[ref26] Gutierrez E. M. Bartonek A. Haglund-Akerlind Y. Saraste H. (2003). Centre of mass motion during gait in persons with myelomeningocele. Gait Posture 18, 37–46. PMID: 14654206 10.1016/s0966-6362(02)00192-3

[ref27] Hrysomallis C. (2011). Balance ability and athletic performance. Sport Med. 41, 221–232. doi: 10.2165/11538560-000000000-00000 21395364

[ref28] Huijgen B. C. H. Elferink-Gemser M. T. Ali A. Visscher C. (2013). Soccer skill development in talented players. Int. J. Sports Med. 34, 720–726. doi: 10.1055/s-0032-1323781, PMID: 23459855

[ref29] Ibrahim R. Faber G. S. Kingma I. van Dieën J. H. (2017). Kinematic analysis of the drag flick in field hockey. Sports Biomech. 16, 45–57. doi: 10.1080/14763141.2016.1182207, PMID: 27192924

[ref30] Ivanenko Y. Gurfinkel V. S. (2018). Human postural control. Front. Neurosci. 12:171. doi: 10.3389/fnins.2018.00171 29615859 PMC5869197

[ref31] Jaeger M. Freiwald J. Engelhardt M. Lange-Berlin V. (2003). Differences in hamstring muscle stretching of elite field hockey players and normal subjects. Sportverletz. Sportschaden 17, 65–70. doi: 10.1055/s-2003-40131, PMID: 12817317

[ref9001] Knudson D. (2007). Qualitative biomechanical principles for application in coaching. Sports Biomech. 6, 109–118. doi: 10.1080/14763140601062567 17542182

[ref33] Kruger M. Lammers M. Stoner L. Allyn D. Fuller R. (1996). Musical expertise: the dynamic movement of the trombone slide. 1996. Eng. Sport

[ref34] Leyhr D. Kelava A. Raabe J. Höner O. (2018). Longitudinal motor performance development in early adolescence and its relationship to adult success: an 8-year prospective study of highly talented soccer players. PLoS One 13:e0196324. doi: 10.1371/journal.pone.0196324 29723200 PMC5933705

[ref9002] Lohse K. R. Sherwood D. E. (2012). Thinking about muscles: the neuromuscular effects of attentional focus on accuracy and fatigue. Acta Psychol (Amst). 140, 236–245. doi: 10.1016/j.actpsy.2012.05.009 22683497

[ref38] Macutkiewicz D. Sunderland C. (2011). The use of GPS to evaluate activity profiles of elite women hockey players during match-play. J. Sports Sci. 29, 967–973. doi: 10.1080/02640414.2011.570774, PMID: 21604228

[ref39] Macutkiewicz D. Sunderland C. (2018). Sodium bicarbonate supplementation does not improve elite women’s team sport running or field hockey skill performance. Physiol. Rep. 6:e13818. doi: 10.14814/phy2.13818 30318837 PMC6186818

[ref41] Maffiuletti N. A. Aagaard P. Blazevich A. J. Folland J. Tillin N. Duchateau J. (2016). Rate of force development: physiological and methodological considerations. Eur. J. Appl. Physiol. 116, 1091–1116. doi: 10.1007/s00421-016-3346-6, PMID: 26941023 PMC4875063

[ref43] Mapelli A. Zago M. Fusini L. Galante D. Colombo A. Sforza C. (2014). Validation of a protocol for the estimation of three-dimensional body center of mass kinematics in sport. Gait Posture 39, 460–465. doi: 10.1016/j.gaitpost.2013.08.025, PMID: 24054347

[ref44] McGuinness A. McMahon G. Malone S. Kenna D. Passmore D. Collins K. (2020). Monitoring wellness, training load, and running performance during a major international female field hockey tournament. J. Strength Cond. Res. 34, 2312–2320. doi: 10.1519/JSC.0000000000002835, PMID: 30216252

[ref45] Meulman H. N. Berger M. A. M. van der Zande M. E. Kok P. M. Ottevanger E. J. C. Crucq M. B. (2012). Development of a tool for training the drag flick penalty corner in field hockey. Pro. Eng. 34, 508–513. doi: 10.1016/j.proeng.2012.04.087

[ref46] Mota J. A. Gerstner G. R. Giuliani H. K. (2019). Motor unit properties of rapid force development during explosive contractions. J. Physiol. 597, 2335–2336. doi: 10.1113/JP277905, PMID: 30919962 PMC6487929

[ref47] Ng L. Rosalie S. M. Sherry D. Loh W. B. Sjurseth A. M. Iyengar S.. (2018). A biomechanical comparison in the lower limb and lumbar spine between a hit and drag flick in field hockey. J. Sports Sci. 36, 2210–2216. doi: 10.1080/02640414.2018.1445440, PMID: 29493427

[ref49] Opala-Berdzik A. Głowacka M. Słomka K. J. (2021). Characteristics of functional stability in young adolescent female artistic gymnasts. J. Hum. Kinet. 77, 51–59. doi: 10.2478/hukin-2021-0051 34168691 PMC8008304

[ref50] Orendurff M. S. Segal A. D. Klute G. K. Berge J. S. Rohr E. S. Kadel N. J. (2004). The effect of walking speed on center of mass displacement. J. Rehabil. Res. Dev. 41, 829–834. doi: 10.1682/jrrd.2003.10.0150 PMID: 15685471

[ref51] Rousanoglou E. N. Herzog W. Boudolos K. D. (2010). Moment-angle relations in the initial time of contraction. Int. J. Sports Med. 31, 651–655. doi: 10.1055/s-0030-1255114 20617488

[ref52] Saito H. Yamanaka M. Kasahara S. Fukushima J. (2014). Relationship between improvements in motor performance and changes in anticipatory postural adjustments during whole-body reaching training. Hum. Mov. Sci. 37, 69–86. doi: 10.1016/j.humov.2014.07.001, PMID: 25108269

[ref53] Seidel O. Carius D. Kenville R. Ragert P. (2017). Motor learning in a complex balance task and associated neuroplasticity: a comparison between endurance athletes and nonathletes. J. Neurophysiol. 118, 1849–1860. doi: 10.1152/jn.00419.2017, PMID: 28659467 PMC5599667

[ref54] Shadmehr R. (2017). Distinct neural circuits for control of movement vs. holding still. J. Neurophysiol. 117, 1431–1460. doi: 10.1152/jn.00840.2016 28053244 PMC5376603

[ref55] Stapley P. J. Pozzo T. Cheron G. Grishin A. (1999). Does the coordination between posture and movement during human whole-body reaching ensure center of mass stabilization? Exp. Brain Res. 129, 134–146. doi: 10.1007/s002210050944 PMID: 10550511

[ref56] Sunderland C. D. Edwards P. L. (2017). Activity profile and between-match variation in elite male field hockey. J. Strength Cond. Res. 31, 758–764. doi: 10.1519/JSC.0000000000001522, PMID: 27359206

[ref9003] Taube W. Gollhofer A. Lauber B. (2020). Training-, muscle- and task-specific up- and downregulation of cortical inhibitory processes. Eur J Neurosci. 51, 1428–1440. doi: 10.1111/ejn.14538 31397937

[ref58] van den Tillaar R. Ettema G. (2006). A comparison between novices and experts of the velocity-accuracy trade-off in overarm throwing. Percept. Mot. Skills 103, 503–514. doi: 10.2466/pms.103.2.503-514 PMID: 17165415

[ref59] Vanderstukken F. Borms D. Berckmans K. Spanhove V. Cools A. M. (2020). Relative scapular-muscle ratios during maximal isokinetic shoulder-girdle strength performance in elite field hockey players. J. Athl. Train. 55, 274–281. doi: 10.4085/1062-6050-499-18, PMID: 31986102 PMC7093928

[ref60] Verburgh L. Scherder E. J. A. Van Lange P. A. M. Oosterlaan J. (2016). The key to success in elite athletes? Explicit and implicit motor learning in youth elite and non-elite soccer players. J. Sports Sci. 34, 1782–1790. doi: 10.1080/02640414.2015.1137344 26788666

[ref63] Wren T. A. L. Do K. P. Rethlefsen S. A. Healy B. (2006). Cross-correlation as a method for comparing dynamic electromyography signals during gait. J. Biomech. 39, 2714–2718. doi: 10.1016/j.jbiomech.2005.09.006 PMID: 16219314

[ref64] Yussof S. Hasan N. Wilson B. (2008). Tree-dimensional biomechanical analysis of the hockey drag flick performed in competition. ISN Bull. 1, 35–43.

